# A causal role of the ventromedial prefrontal cortex in regulating anticipated regret and risk behaviors in trait anxiety

**DOI:** 10.1017/S0033291726103882

**Published:** 2026-06-01

**Authors:** Hui Ai, Mengting Wang, Mengli Zhang, Ying Zeng, Jingyi Gao, Lin Huang, Lian Duan, Pengfei Xu, Li An

**Affiliations:** 1 Laboratory of Suicidal Behavior Research, Institute of Applied Psychology, Tianjin University, Tianjin, China; 2 School of Education, Tianjin University, Tianjin, China; 3 Shenzhen Key Laboratory of Affective and Social Neuroscience, Center for Brain Disorders and Cognitive Sciences, Shenzhen University, Shenzhen, China; 4 Beijing Key Laboratory of Applied Experimental Psychology, National Demonstration Center for Experimental Psychology Education (BNU), Faculty of Psychology, Beijing Normal University, Beijing, China; 5 Faculty of Health and Wellness, City University of Macau, Taipa, Macau

**Keywords:** anxiety, counterfactual-thinking, decision-making, HD-tDCS, regret

## Abstract

**Background:**

Individuals with high trait anxiety (HA) exhibit maladaptive goal-directed behaviors, which are associated with dysfunctional counterfactual-thinking during decision-making. While lesion studies suggest the causal role of the ventromedial prefrontal cortex (vmPFC) in counterfactual-thinking, its modulatory role in anxiety-related counterfactual decision-making remains uncharacterized. Here, we bridge this gap by examining the characteristics of decision-making (forward counterfactual) and emotion responses (backward counterfactual) in trait anxiety, as well as its underlying modulatory mechanisms by targeting at the vmPFC.

**Methods:**

A counterfactual-thinking paradigm was employed to identify the patterns of goal-directed choice and emotional responses in trait anxiety in experiment 1. In all, 107 participants with varied levels of trait anxiety were recruited and counterfactual indices were modeled. In experiment 2, the high-definition transcranial direct current stimulation (HD-tDCS) was applied to modulate forward and backward counterfactual responses targeting at the vmPFC in HA. Based on the exploratory results of experiment 1, 61 participants with HA were randomly assigned to cathodal or sham stimulation.

**Results:**

High level of anxiety was associated with stronger emotional responses to backward counterfactuals, more anticipations of regret to forward counterfactuals, higher value-expectations to potential rewards, and more risk-taking behaviors. Related to sham, cathodal HD-tDCS over the vmPFC in HA showed normalized sensitivity to anticipated regret, which leads to less risk-taking behaviors during goal-directed decision-making.

**Conclusions:**

The findings provide evidences of disrupted forward and backward counterfactual processing in anxious individuals, wherein the vmPFC plays a modulatory role. Targeting vmPFC with noninvasive stimulation may normalize maladaptive decision patterns in anxiety and anxiety disorders.

## Introduction

One of the most prevalent characteristics of anxiety is an excessive or disproportionate concern about the future (Newman et al., 2013). According to the Uncertainty and Anticipation Model of Anxiety (UAMA), individuals with anxiety are inclined to overestimate the probability of unfavorable outcomes in uncertain situations (Geng et al., 2018). This cognitive bias can result in an excessively pessimistic outlook and the emergence of maladaptive behavioral responses (Grupe & Nitschke, 2013). Furthermore, the overestimation of the likelihood of negative events also gives rise to heightened negative emotions and further impairs decision-making (Carleton, 2016; Gagne et al., 2018).

The evaluation of past outcomes and the formulation of future expectations are the fundamental elements of the decision-making process (Howlett & Paulus, 2013), which is influenced by counterfactual alternative outcomes after the counterfactual thinking (Li et al., 2022). Counterfactual thinking is characterized by the utilization of mental simulation and cognitive reasoning skills to contemplate alternative scenarios or potential outcomes (Roese, 1997). When the reality is evaluated in comparison to hypothetical alternatives, upward counterfactual processing gives rise to feelings of disappointment and regret when individuals imagine that the potential outcome is superior to the actual outcome. In contrast, downward counterfactual processing gives rise to positive feelings such as pleasure and gratitude when individuals envision a potential outcome that is perceived to be inferior to reality (Markman et al., 2006). The cognitive process of counterfactual thinking along with its associated emotional impact, not only facilitate individuals’ goal-oriented behaviors but also enhances their sense of control over future events (Roese & Epstude, 2017). Therefore, an investigation into the processing of counterfactual thinking in individuals with anxiety can facilitate the understanding of the emotional and decision-making mechanisms that underpin anxiety, thereby offering insights into potential interventions.

The evidence from neuroimaging studies indicates that counterfactual thinking involves the activities of various brain networks, including the reward network, the cognitive control network, and the default network (Tagini et al., 2021). Within these networks, the ventromedial prefrontal cortex (vmPFC) is a core node and plays a crucial role in the representation of reward-based decision-making, the generation and regulation of negative emotion, and social cognition (Hiser & Koenigs, 2018). Patients with lesions in the vmPFC have been reported to exhibit deficits in value-based decision-making, struggle to modify their expectations flexibly, and display diminished levels of disappointment or a lack of regret (Bault et al., 2019; Levens et al., 2014; Messimeris et al., 2023). The causal role of vmPFC on value-based decision-making and emotion regulation indicates that it could be a potential intervention target. However, it remains unclear whether modulating the vmPFC would change the cognitive and emotional responses during counterfactual decision-making in individuals with anxiety.

Therefore, the aim of the study was to understand the emotional and cognitive mechanisms underlying the processing of counterfactual-thinking in trait anxiety. A previous study indicates that self-report methods of counterfactual thinking may be influenced by memory biases related to comorbid anxiety and depression (Ho et al., 2018). It is critical to examine whether the processing of counterfactual cognition and emotion is altered in anxiety using objective and quantitative methodologies. Computational modeling quantifies symptoms into basic neuro-computational mechanisms and may help to elucidate the decision-making processing influence by levels of anxiety (Calder et al., 2018). Therefore, we employed an adapted version of the Economic Decision-Making task and used computational modeling to investigate the reward anticipation, risk-aversion, and regret anticipation during the decision-making process in individuals with trait anxiety.

Previous studies have indicated that anxious individuals showed increased susceptibility to emotional stimuli (Rutter et al., 2021) and diminished capacity for emotional regulation in the presence of negative events (Nook et al., 2017). Therefore, we predicted that individuals with HA may experience stronger counterfactual emotions compared to those with low trait anxiety. Moreover, individuals with anxiety have been reported to be risk-averse (Charpentier et al., 2017; Giorgetta et al., 2012) and have more avoidance behaviors to cope with the uncertainty of potential threats to enhance their sense of control over the future (Grupe & Nitschke, 2013). So, we hypothesized that individuals with HA would be more risk-aversive and more sensitive to anticipated regret through counterfactual thinking compared to those with low trait anxiety. Furthermore, we aimed to explore the role of the vmPFC in goal-directed choice and emotional responses in trait anxiety using high-definition transcranial direct current stimulation (HD-tDCS). Compared to traditional tDCS, HD-tDCS not only possesses the excellent features of being noninvasive, safe, and comfortable, but it also provides higher spatial accuracy. This enables a precise stimulation targeted at both the cortical and subcortical regions of interest (Sergiou et al., 2022).

## Experiment 1

### Method

#### Participants

A total of 107 college students, including 46 males and 61 females, were recruited for this experiment. Trait anxiety was measured by The State Trait Anxiety Inventory (Shek, 1993). Severity of depression and level of impulsivity were measured by The Beck Depression Inventory Second Edition (BDI-II; Beck et al., 1996) and The Barratt Impulsiveness Scale-11 (BIS-11; Patton et al., 1995), respectively. According to Takacs et al. (2015), we ranked the scores on the trait anxiety scale from highest to lowest, with subjects obtaining scores in the top 50% being in the high-anxiety group, and subjects obtaining scores in the bottom 50% being in the low-anxiety (LA) group. Moreover, we included impulsivity as a covariate because it has been consistently linked to increased risk-taking behaviors independent of risk perception, suggesting its role as a direct influence on decision processes under uncertainty (Megías-Robles et al., 2022). To ensure a comparable level of impulsivity across the two groups, the three subjects with the highest impulsivity scores in the high-anxiety group and the three subjects with the lowest impulsivity scores in the LA group were excluded. One subject did not complete the experiment and was thus excluded from subsequent analyses. These resulted in 50 subjects in the high-anxiety group (HA, 20 males, mean age = 20.14, SD = 1.59) and 50 subjects in the low-anxiety group (LA, 24 males, mean age = 19.82, SD = 1.37).

Prior to the experiment, each participant provided informed consent through a signed consent form. The study protocol was approved by the University’s Institutional Review Board and was in accordance with the principles outlined in the Declaration of Helsinki. Table 1 shows the demographic characteristics.

**Table 1.** Descriptive statistics and scores on each scale (M ± SD) for the high and low anxiety groups in Experiment 1 and Experiment 2 Table 1. long description.

Variants	Experiment 1				Experiment 2			
	HA	LA	*t*/χ^2^	*p*	HAC	HAS	*t*/χ^2^	*p*
Sex (M/F)	20/30	24/26	0.65	0.42	13/17	16/15	0.56	0.52
Age	20.14 ± 1.59	19.82 ± 1.37	−1.08	0.28	19.90 ± 1.58	20.61 ± 2.16	−1.47	0.15
BDI	11.42 ± 7.41	3.16 ± 2.96	−7.32	< 0.001	14.53 ± 7.95	15.19 ± 6.65	−0.35	0.73
SAI	43.90 ± 9.52	30.58 ± 5.74	−8.48	< 0.001	44.70 ± 5.82	45.19 ± 9.56	−0.24	0.81
TAI	48.80 ± 6.70	33.34 ± 4.43	−13.61	< 0.001	53.80 ± 4.65	54.94 ± 5.05	−0.91	0.37
BIS-11	58.18 ± 8.39	56.08 ± 7.31	−1.34	0.19	60.53 ± 8.18	62.61 ± 9.84	−0.90	0.37

*Note*: HAC, High anxiety cathodal stimulus group; HAS, High anxiety sham stimulus group; BDI, Beck Depression Inventory score; SAI, State Anxiety Inventory score; TAI, Trait Anxiety Inventory score; BIS-11, Impulsivity Inventory score.

#### Task design

We employed an economic decision-making task developed by Gillan et al. (2014) (see Supplementary Figure S1). Participants were instructed to choose between two wheels, each showing gains and losses with their probabilities. In half of the trials, participants were allowed to change their mind. Once a choice has been made, the outcome of the chosen wheel will be displayed, and the participants were instructed to rate their emotional responses (rating 1: partial feedback). Following the initial rating made by participants during the partial feedback phase, the results of the wheel that was not selected will be displayed on the screen. Subsequently, participants will be required to provide a second emotional rating for the outcome obtained (rating 2: complete feedback).

#### Data analyses

***Affective responses.*** A linear mixed-effects model (LMM) was built using the lme4 package in R (version 4.0.5) to analyze affective responses. In this model, the group (HA, LA) was set as a fixed effect factor, and subject as a random effect factor. Separate analyses were conducted for rating 1 and rating 2.

For partial feedback, we modeled the effects of the value of the obtained outcome and the chance counterfactual (i.e., the difference between what was obtained and what could have been obtained from the selected wheel) on their affective responses.

For complete feedback, we modeled the effects of the value of the obtained outcome and the agent counterfactual (i.e., the difference between what was obtained for the selected wheel and what could have obtained from the unselected wheel) on the affective responses.

The differences between affective responses in partial and complete feedback under the condition of relative gain and loss were also examined. Owing to non-normality of the affective rating scores, a Friedman’s ANOVA was applied to examine the main effect of condition (relative gain and loss) on ratings per group (*p* < 0.05). Post hoc analyses were performed with a Wilcoxon signed-rank test (*p* < 0.013, Bonferroni correction for four tests). To examine group differences on the rating scores, Mann–Whitney *U* tests were performed (*p* < 0.013, Bonferroni correction for four tests).

To assess the effect size of the interaction effect within the affective rating model, we utilized Cohen’s ƒ^2^, a statistic that is derived by evaluating the difference in *R* ^2^ values between models that include the interaction term and those that do not (ƒ^2^ = Δ*R* ^2^ /(1−*R* ^2^)). This method provides a more precise assessment of the contribution of interaction to the model’s explanatory power, enabling researchers to identify the importance of the interaction beyond mere statistical significance (Selya et al., 2012).

***Decision-making modeling.*** The effect of three parameters, expected value (EV), risk variance (V), and anticipated regret (R), on decision-making behavior were modeled.

The following parameters were defined: the values of *x*1 and *y*1 referred to the two potential outcomes of wheel 1 (W1), while *x*2 and *y*2 represented the two potential outcomes of wheel 2 (W2). It was assumed that *x*1 > *y*1 and *x*2 > *y*2. The probabilities of obtaining *x*1 and *y*1 were denoted by *p* and 1 - *p*, respectively, while the probabilities of obtaining *x*2 and *y*2 were denoted by *q* and 1–*q* (see Supplementary Figure S2).

The expected value (EV) was obtained by subtracting the expected value of W2 from the expected value of W1. When EV > 0, participants seeking to choose the wheel with the greater EV should choose W1. EV was calculated using the following formula:

(1) $$\mathrm{EV}=EV_{W1}-EV_{W2}=\left[p*x_{1}+\left(1-p\right)*y_{1}\right]-\left[q*x_{2}+\left(1-q\right)*y_{2}\right]$$

The risk variance (*V*), compares the relative variances of two wheels. It was calculated by subtracting the risk of W1 from the risk of W2. If *V* > 0, it indicated that W2 carries a higher risk than W1. To avoid risk, a person should opt for W1. The risk of W1 (VW1) and the risk variance (*V*) were calculated as follows:

(2) $$V_{W1}=p*{\left(x_{1}-EV_{W1}\right)}^{2}+\left(1-p\right)*{\left(y_{1}-EV_{W1}\right)}^{2}$$

(3) $$\begin{array}{c} V=V_{W2}-V_{W1}=\left[q*{\left(x_{2}-EV_{W2}\right)}^{2}+\left(1-q\right)*{\left(y_{2}-EV_{W2}\right)}^{2}\right]\\ -[p*(x_{1}{-\;EV_{W1})}^{2}+\left(1-p\right)*{\left(y_{1}-EV_{W1}\right)}^{2}] \end{array}$$

In order to assess the discrepancy between the least favorable outcome of one wheel and the most favorable outcome of the other, it is necessary to consider the difference between the obtained outcome and the possible outcome. This difference determines the degree of regret and gratitude experienced (*R*). In scenarios where *r* > 0, the individual aiming to minimize anticipated regret should opt for W1. The formula was delineated as follows:

(4) $$R=\left(y_{1}-x_{2}\right)-\left(y_{2}-x_{1}\right)$$

The probability of choosing wheel 1 (*P*_(*W*1it)_) was calculated, where *t* referred to the number of trials and *i* denotes the participant. The calculation formula of *P*_(*W*1*it*)_ is as follows:

(5) $$P_{\left(W1it\right)}=1-P_{\left(W2it\right)}=F\left(e_{\mathrm{it}},v_{\mathrm{it}},r_{\mathrm{it}}\right)$$

(6) $$F\left(\theta \right)=e^{\theta }*\left(1+e^{\theta }\right)$$

F is the inverse function of the logistic function and *θ* is the value predicted by EV, *V*, and *R* in logistic regression.

The logistic regression was used to test the main effect and interactions between model parameters and group. And the likelihood ratio tests were used to confirm the statistical significance of the model. Finally, Cohen’s ƒ^2^ was also calculated to evaluate the power of the models. Because of the high comorbidity with anxiety and depression, the level of depression was also different between HA and LA. Therefore, to control for the effect of depression, we repeated the analyses after matching the level of depression between groups. State anxiety was not included as a covariate because it represents a situational manifestation of trait anxiety and is therefore not independent of the construct under investigation.

### Results

#### Affect ratings

**Figure 1.**
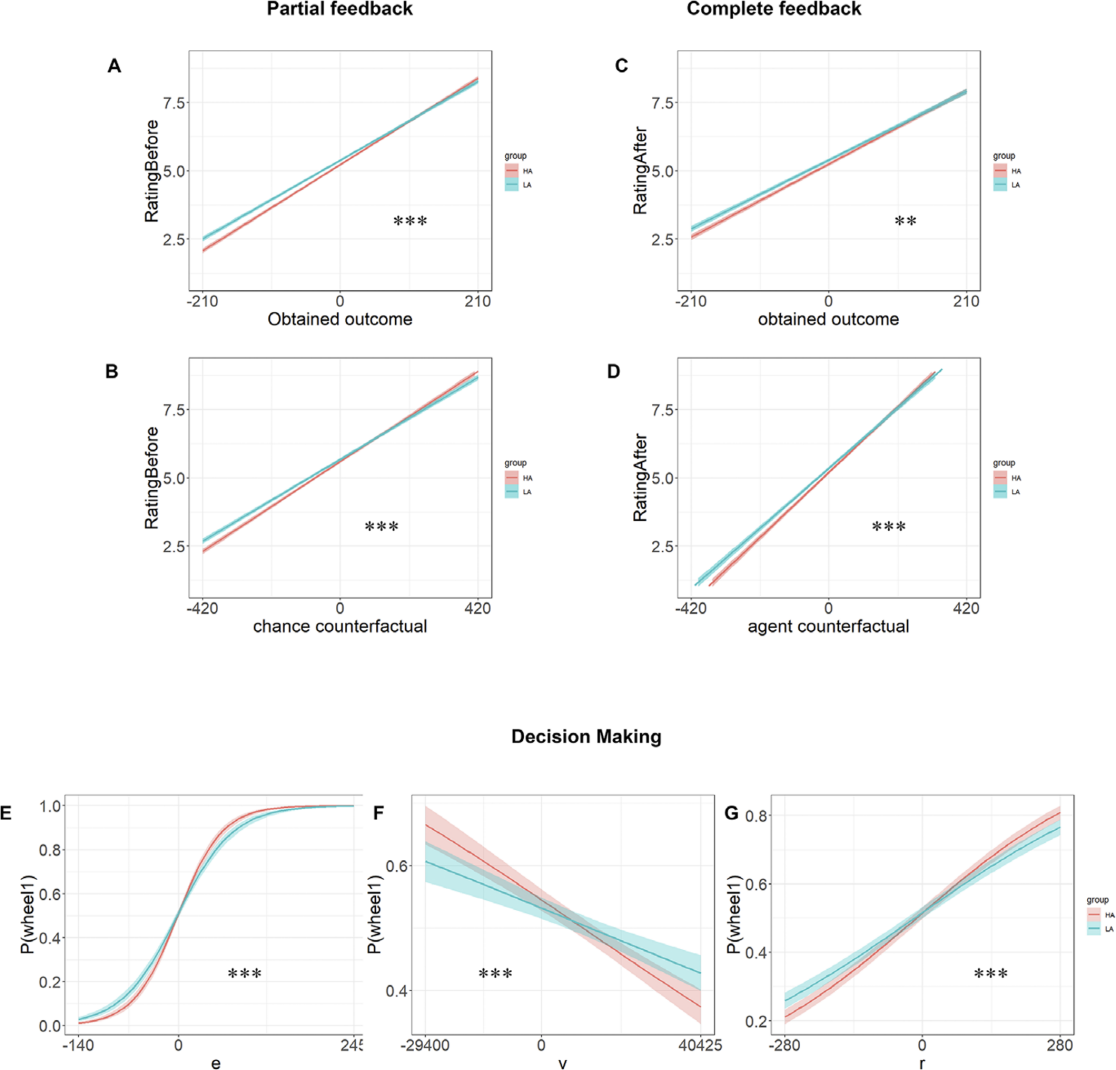
Affective ratings for partial and complete feedback and behavioral sensitivity to regret, expected value, and risk variance in high and low anxiety (Experiment 1). The panels show that effect of (a) obtained outcome and (b) chance counterfactual affect rating following partial feedback; and the effect of (c) obtained outcome and (d) agent counterfactual on affect rating following complete feedback; The logit model-predicted probability of choosing wheel 1 at varying levels of (e) expected value, (EV) and (f) risk variance (*V*), (g) anticipated regret (*R*). HA = high anxiety group; LA = low anxiety group. *p < 0.05, **p < 0.01, ***p < 0.001, NS = nonsignificant. Figure 1. long description.

During the partial feedback, there was a significant interaction between group and obtained outcome (*p* < 0.001, ƒ^2^ = 0.21 × 10^−2^; Figure 1a), with more extreme affective responses in HA being more strongly influenced by the obtained outcome compared to LA. A significant interaction between group and chance counterfactual was also observed (*p* < 0.001, ƒ^2^ = 0.19 × 10^−2^), with HA showing more extreme affective responses compared to LA (Figure 1b). Furthermore, there were significant main effects of obtained outcome and chance counterfactuals across all participants (*ps* < 0.001). After matching the level of depression between the two groups, the interaction between group and obtained outcome was still statistically significant (*p* = 0.002, ƒ^2^ = 0.82 × 10^−3^), as well as the interaction between group and chance counterfactual (*p* = 0.008, ƒ^2^ = 0.74 × 10^−3^).

During the complete feedback, a significant interaction was also observed between group and obtained outcome (*p* = 0.003, ƒ^2^ = 0.09 × 10^−2^; Figure 1c). The groups with high trait anxiety were more affected by the obtained outcome compared to those with low trait anxiety. Additionally, there was a significant interaction between group and agent counterfactual (*p* < 0.001, ƒ^2^ = 0.15 × 10^−2^; Figure 1d). The affective responses of individuals in both groups were influenced by the unobtained outcome, and the group with HA experienced greater gratitude when the agent counterfactual was large and more regret when it was small, as opposed to those with low trait anxiety. The main effects of the obtained outcome and the agent counterfactual were also significant (*ps* < 0.001). After matching the level of depression between HA and LA, the interaction between the group and obtained outcome was no longer statistically significant (*p* = 0.74), nor was the interaction between group and agent counterfactual (*p* = 0.60).

For the affective responses on relative gain and loss, significant main effects of group and condition were observed (*p* < 0.05). Post hoc analyses showed that HA was more negative than LA under the condition of relative loss during partial feedbacks (*p* < 0.001) as well as complete feedbacks (*p* < 0.001). For the condition of relative gain, HA had more positive ratings than LA only during partial feedbacks (*p* < 0.001) but not during complete feedbacks (*p* = 0.16).

#### Decision-making

Group differences in the degree to which the avoidance of regret (*R*) and risk (*V*) and the promotion of expected value (EV) predicted choice behaviors were tested by the interactions between group and the decision-making parameters. Significant effects of interactions were found between group and EV, *V* and *R* (*ps* < 0.001,ƒ^2^ = 0.30) (see Supplementary Table S1), with EV, *V*, and *R* as stronger predictors of wheel choice in the HA group compared with the LA group (Figure 1). Significant main effects were also found for EV, *V*, and *R* (*ps* < 0.001).

After controlling for the level of depression between groups, the interactions between *V* and group (*p* = 0.04) and between R and group (*p* < 0.001,ƒ^2^ = 0.01) remained significant, whereas the interaction between EV and group was not significant (*p* = 0.62) (see Supplementary Table S2).

#### Correlation analysis of primary variables

To explore the correlations between emotional/decision-making parameters and anxiety levels, we carried out correlation analyses on the principal task variables and the corresponding scale scores in individuals with HA. Although correlations were found within the task variables, there were no significant correlations between task variables and demographic variables, level of depression and anxiety, and level of impulsivity (Supplementary Table S4).

Overall, we observed distinctive patterns of backward counterfactual and forward counterfactual processing during decision-making in individuals with HA. First, the HA group showed stronger affective responses to chance counterfactuals compared to the LA group, even after controlling for the level of depression. Affective responses to agent counterfactuals were also stronger in HA, but this may be influenced by depressive states, because the effect was not significant after controlling for the level of depression. Second, the avoidance of future regret was increased in HA, while the avoidance of risk was attenuated in HA compared to the LA group. These effects were not influenced by level of depression. The expected value was also greater in the HA group than the LA group in the model, but it contributed equally in the two groups after controlling for the level of depression. Based on these, to modulate the counterfactual processing in anxiety, it is important to suppress their backward counterfactuals and downregulate their avoidance of regret.

## Experiment 2

### Methods

#### Participants

To better capture the characteristics of trait anxiety, we ranked the scores on the STAI-T scores of 242 participants from highest to lowest and defined those scoring in the top 27% as the HA group. In addition, to ensure that participants we included were likely to have clinically meaningful anxiety symptoms, the HA group should have above a threshold score of 48 (Heffer et al., 2022). These resulted in a total of 61 participants in study 2, including 29 males and 32 females. They were randomly assigned into the cathodal group (HAC, *n* = 30) and the sham group (HAS, *n* = 31). Table 1 shows the demographic characteristics of the participants.

#### High-definition transcranial direct current stimulation (HD-tDCS) settings

While anodal tDCS depolarizes the membrane potential of underlying neurons and increases their excitability, a cathodal stimulation hyperpolarizes the membrane potential and diminishes the neurons’ excitability (Sparing & Mottaghy, 2008). Given the key role of the vmPFC in emotion regulation and decision-making (Hiser & Koenigs, 2018) and hyperactivation in the mPFC in trait anxiety (Hein et al., 2023), we applied a cathodal stimulation targeting this area to decrease the excitability so as to interevent the altered backward and forward counterfactual processing in individuals with high anxiety (Jafari et al., 2021). Moreover, compared to conventional montages, HD configurations allow for more focal and longer-lasting diffusion of the current. Therefore, experiment 2 was designed as a double-blind, placebo-controlled, randomized experiment by comparing a cathodal HD-tDCS group with a sham control group.

**Figure 2.**
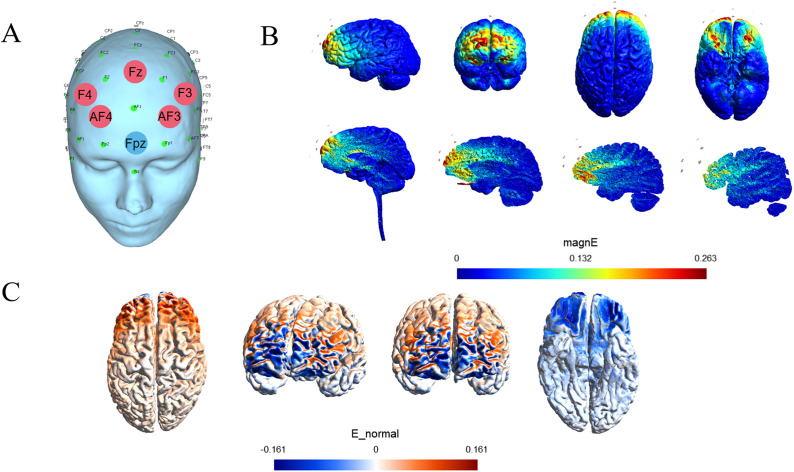
Electrical field model targeting the vmPFC created by Gmsh (V4.7.1). (a) Stimulation sites for cathodal HD-tDCS stimulation of the vmPFC. The red circles (AF3, AF4, F3, F4, Fz) indicate the stimulation sites of the return electrode with a current intensity of 400 μA, while the red circles indicate the stimulation sites of the target electrode (Fpz) with a current intensity of −2 mA. (b) Different views and slices of the electric field maps induced by HD-tDCS are expressed in magnE. Colors closer to red indicate greater current intensity flowing through the region. (c) Plot of the electric field distribution of the current polarity expressed as E_normal. Negative values (blue) indicate cathode, and positive values (red) indicate anode. Figure 2. long description.

HD-tDCS was delivered by a battery-powered, wireless multichannel transcranial current stimulator (NeuStim NSS18, Neuracle, China), and six circular Ag/AgCl high-definition electrodes (5 × 1 montage, five return electrodes and one target electrode) were applied using conductive gel. According to the International 10–20 EEG system and previous studies (Sergiou et al., 2022), the center electrode was positioned over Fpz, and the other five return electrodes over F4, AF4, Fz, F3, and AF3 (see Figure 2a for the electrical field model). For the cathodal condition, 2 mA current was transmitted for 20 minutes with a 30-second ramping-up and down. Participants commenced the tasks after 8 minutes of stimulation. For the sham condition, the electrical stimulation was transmitted only during the first and final 30 seconds, with the rest of the settings identical to those of the cathodal stimulation condition. The 30 s period of current stimulation elicited a tingling sensation on the scalp that was similar to that experienced in the cathodal group. However, it did not impact neural activity during the formal task (Feeser et al., 2014; Riva et al., 2015). All participants in study 2 reported scalp itching at the onset of stimulation but no other adverse effects. All participants confirmed that they had received electrical stimulation throughout the task.

#### Data analysis

An LMM was employed using the lme4 package in *R* (version 4.0.5) to analyze affective responses similar to analyses conducted in experiment 1. In this model, we set the group (HAC, HAS) as a fixed effect factor, and subject as a random effect factor. Two distinct analyses were conducted, one for rating 1 (following partial feedback) and another for rating 2 (following complete feedback). The effect of condition (relative gain and loss) and the effect of group were also examined as experiment 1.

Furthermore, the effects of three parameters, namely expected value (EV), risk variance (*V*), and anticipated regret (*R*), on decision-making behavior were evaluated by the same computational models as in experiment 1.

### Results

#### Affect ratings

**Figure 3.**
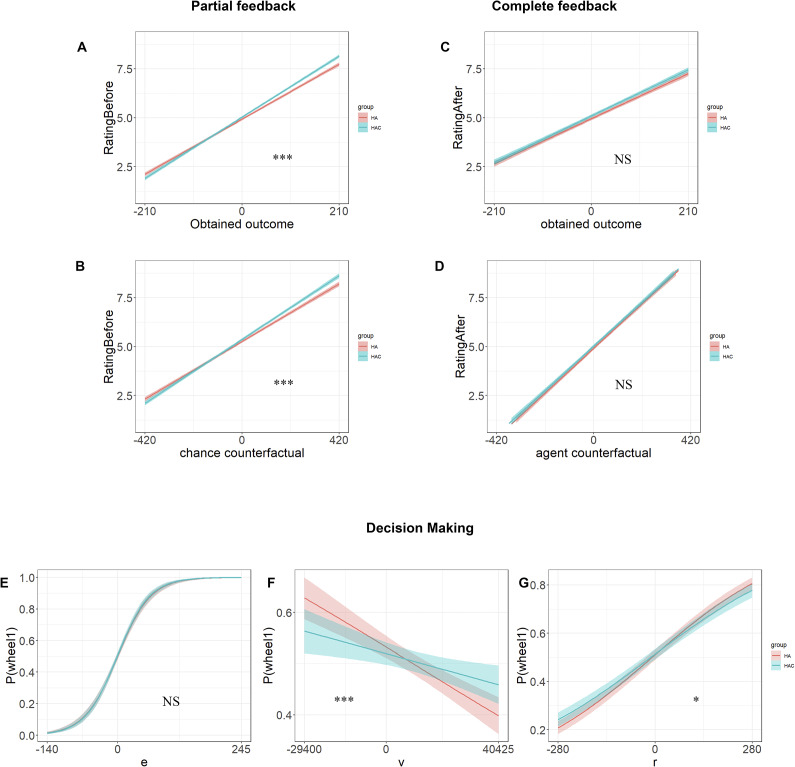
Affective ratings for partial and complete feedback and behavioral sensitivity to regret, expected value, and risk variance after cathodal HD-tDCS at the vmPFC (Experiment 2). The panels show that effect of (a) obtained outcome and (b) chance counterfactual affect rating following partial feedback; and the effect of (c) obtained outcome and (d) agent counterfactual on affect rating following complete feedback. The logit model-predicted probability of choosing wheel 1 at varying levels of (*E*) expected value (EV), (f) risk variance (*V*), and (g) anticipated regret (*R*). HA = high anxiety group; LA = low anxiety group. *p < 0.05, **p < 0.01, ***p < 0.001, NS = nonsignificant. Figure 3. long description.

During the partial feedback, a significant interaction was observed between type of group and obtained outcome (*p* < 0.001; ƒ^2^ = 0.27 × 10^−2^; Figure 3a), indicating that the cathodal stimulation group was more influenced by the obtained outcome after stimulation compared to the sham stimulation group (*p* < 0.001). Furthermore, the interaction between the type of group and the chance counterfactual was also significant (*p* < 0.001;ƒ^2^ = 0.29 × 10^−2^; Figure 3b); the cathodal group was more sensitive to the chance counterfactual after stimulation compared to the sham group (*p* < 0.001). There were significant main effects for the obtained outcome and the chance counterfactual across all participants (*p* < 0.001) (Figure 3a,b, indicating that a higher value of the obtained outcome and chance counterfactual was associated with more positive ratings. The main effect of the group was not statistically significant (*p* = 0.29). In summary, the cathodal group showed greater affective reaction to the value whether they won or lost.

During the complete feedback, the main effects of the obtained outcome and the agent counterfactual were statistically significant (*p* < 0.001) (Figure 3c; Figure 3d). The main effect of the group was not statistically significant (*p* = 0.26), nor were any interactions between the group and obtained outcome (*p* = 0.39; Figure 3c), or between the group and agent counterfactual (*p* = 0.83; Figure 3d).

For the relative gain condition, the cathodal group showed more positive responses than the sham group during partial feedback (*p* = 0.007) but not during complete feedback (*p* = 0.85). Group differences were not significant with respect to relative loss during partial or complete feedbacks (*ps* > 0.013).

#### Decision-making

Significant interactions were observed between the group and *V*, group and R (*p* < 0.001; ƒ^2^ = 0.80 × 10^−2^; see Supplementary Table S3), with the cathodal group being less sensitive to *R* and *V* after stimulation compared to the sham group (Figure 3f; Figure 3g). Significant main effects were also found for the parameters EV, *V*, and *R* (*p* < 0.001). The interaction between the group and EV was not significant (*p =* 0.68).

#### Correlation analysis of main variables

Correlation analyses were conducted on the principal task variables and the corresponding scale scores. The results, as presented in Supplementary Table S5, indicated a notable correlation among the task variables and no correlation between task variables and demographic factors, scores on the depression, trait anxiety, or impulsivity.

## Discussion

The present study examined the behavioral and neural mechanisms of counterfactual decision-making in trait anxiety using a mathematical model of choice and HD-tDCS. A high level of trait anxiety was associated with increased emotional responses after backward counterfactual-thinking, especially under the condition of loss. Altered patterns in goal-directed modeling after forward counterfactual thinking were also found in individuals with HA, observed using a mathematic model of choice; these individuals relied excessively on the avoidance of future regret and on less risk-aversion to guide them in decision-making. The altered pattern of emotional and cognitive processing was independent of the level of comorbid depression. Cathodal stimulation over the vmPFC led to less avoidance of future regret in individuals with high anxiety and normalized their risk-taking behaviors during goal-directed decision-making. Moreover, participants after cathodal stimulation showed increased positive responses than the sham group, especially during partial feedbacks. Sensitivity analyses confirmed that key behavioral patterns remained directionally consistent across different thresholds, supporting the robustness of our findings.

### Counterfactual cognitive responses in anxiety: regret avoidance and risk preference

Individuals with HA relied more on regret minimization than expected value maximization during forward counterfactual decision-making. Previous research has reported more negative counterfactual thinking and lower ratings for negative mental simulations in anxious individuals (Parikh et al., 2020). More frequently thinking about the future involving unimportant and less realistic aspects has been found in patients with generalized anxiety disorder; this serves as a way to keep themselves occupied with some level of awareness of perceived costs and benefits and also helps with boredom reduction (Hallford et al., 2024). Added to this, people with an elevated level of anxiety might engage in forward counterfactual thinking for the purpose of avoiding future regret, so as to protect themselves from painful consequences (Zeelenberg et al., 2000). On the other hand, sensitivity to reward expectation was also observed in individuals with HA but was not independent of depression. This pattern implies that reward sensitivity may not be unique to anxiety but rather reflects a shared mechanism driven by comorbid depressive symptoms. This finding aligns with prior evidence that links depression to altered sustained reward sensitivity (Berry et al., 2019). Moreover, due to the critical role of the vmPFC in strengthening the association between anticipated emotions and choices (Levens et al., 2014), we speculate that individuals with HA may show altered activation in the vmPFC and are inclined to avoid anticipated regret.

Individuals with HA exhibited reduced risk aversion during decision-making in comparison to those with low trait anxiety, which stands in contrast to our hypothesis and to previous views of anxiety being a risk-averse state (Charpentier et al., 2017; Giorgetta et al., 2012). Although anxiety is often associated with risk aversion in description-based economic tasks, it has also been reported that this effect is context-dependent (Gu et al., 2017; Notebaert et al., 2016) and modulated by task structure and uncertainty level (Maner & Schmidt, 2006; Smith et al., 2016). For example, Gu et al. (2017) posited that the association between trait anxiety and risk-aversion can be modulated by the emotional context, suggesting that anxiety influences risk preferences by enhancing sensitivity to negative emotions. Moreover, some researchers have contended that anxiety levels can impact the decision-maker’s cognitive resource allocation (Notebaert et al., 2016), resulting in a reduction of the cognitive resources allocated to information processing by decision-makers (Yanying et al., 2019). Consequently, in contexts necessitating the integration of reward expectations and counterfactual valuations under uncertainty, the discrepancy between our findings and those of other studies may be attributable to the distorted value computation in individuals with HA. This suggests that anxious individuals may be influenced by a reduction or an over-allocation of both emotional and cognitive resources, which can result in biased decision-making and an increased propensity for high-risk choices.

### Counterfactual affective responses in anxiety: enhanced emotional reactivity

Individuals with HA exhibited heightened emotional responses following backward counterfactual thinking, particularly under loss conditions. This is consistent with previous findings of overweighting of loss and lower emotional thresholds in healthy populations with a high level of anxiety (Rutter et al., 2021; Xu et al., 2020). Using ecological transient assessment, Heller et al. (2019) observed that trait anxiety is associated with instability of both positive and negative emotions, suggesting that those with HA may exhibit more extreme emotional responses. Anxious individuals are inclined to interpret ambiguous stimuli or situations as potential threats and are thus more likely to experience heightened happiness upon winning and increased disappointment upon losing (Wilson et al., 2006). Moreover, the affective responses to change counterfactuals were anxiety dependent and the affective responses to agent counterfactuals were shared with depressive symptoms. Consequently, our study suggests that individuals with HA may be more prone to overreacting to immediate outcomes and chance counterfactual due to their lower emotional response thresholds, resulting in a more intense experience of delight and disappointment compared to those with low trait anxiety. In contrast to Zeelenberg et al.’s (2000) proposal that counterfactual thinking serves as an emotion-regulation tool in healthy individuals, our results suggest that high-anxiety individuals may not alleviate emotional burdens through counterfactual reflection. Instead, they tend to engage in “downward counterfactuals” (i.e., imagining worse outcomes), which exacerbates emotional distress. This discrepancy may stem from anxiety-related hyperfocus on potential threats (Wilson et al., 2006), leading counterfactual processing to prioritize negative scenarios over constructive reappraisal.

### Counterfactual neural underpinning in anxiety: the vmPFC as a hub

The vmPFC has been involved in assessing the value of potential outcomes and regulating emotional responses (Fellows & Farah, 2007; Hiser & Koenigs, 2018; Suzuki & Tanaka, 2021). Our HD-tDCS targeting the vmPFC demonstrated that cathodal stimulation reduced regret avoidance in high-anxiety individuals and normalized risk-taking behavior. This directly supports the vmPFC’s role as a critical hub for emotion–decision coupling (Hiser & Koenigs, 2018). Specifically, hyperactivity in the vmPFC may amplify anxiety-related imagination of unrealized outcomes, while cathodal stimulation (which suppresses cortical excitability) weakens this amplification.

Research has indicated that stimulation of the prefrontal cortex with tDCS can significantly improve emotion-regulation abilities in individuals with anxiety (Heeren et al., 2017; Ironside et al., 2016; Kenney-Jung et al., 2019). However, our research shows limited effects of HD-tDCS on emotional regulation. The stimulation did not alter the emotional response of high-anxiety individuals to agent counterfactual outcomes, suggesting that the subjective emotional experience in complete feedback may depend on distributed affective circuits involving regions including the vmPFC, dlPFC, and amygdala (Buhle et al., 2014; Ochsner et al., 2012). Targeting the vmPFC alone may be insufficient due to its dependence on other brain regions (Morawetz et al., 2017). Furthermore, individual brain and cognitive differences (Kanai & Rees, 2011; Seghier & Price, 2018), stimulation parameters such as current intensity, electrode placement, and type of current (Harty & Cohen Kadosh, 2019), or differences in task design (Hill et al., 2017) may also further limit its comprehensive impact on emotional processing.

Together, our results indicate a modulatory role of the vmPFC in normalizing maladaptive behavior even when emotional responses remain elevated. This dissociation may suggest that vmPFC stimulation primarily affects the translation of counterfactual signals into behavioral implementation, whereas the subjective emotional experience of complete feedback may depend on distributed affective circuits.

### Limitations

While this study advances understanding of trait anxiety’s role in counterfactual decision-making, there are some limitations. Firstly, because trait anxiety is inherently dimensional, a continuous construct is necessarily discretized by group-based analyses. Future studies with larger samples will be better positioned to characterize nonlinear effects across the full anxiety spectrum. Replication in clinical anxiety patients is necessary to increase the generalizability of the present findings. Secondly, the employment of computational models facilitated the objective quantification and assessment of counterfactual emotions and decision-making dynamics within the experimental design. It is recommended that future research endeavors incorporate additional objective indicators for monitoring emotional activation, including galvanic skin response and cortisol levels. Thirdly, manipulation through the counterfactual paradigm and the HD-tDCS stimulation were used to measure the short-term behavioral and neural outcomes. Further studies should combine the short-term and long-term assessment to test the follow-up effects.

## Conclusion

Our study, combining behavioral modeling, neural stimulation, and emotion assessment, reveals that individuals with high anxiety traits exhibit amplified emotional responses, increased regret avoidance, and atypical risk preferences in counterfactual thinking contexts. Furthermore, the study supports a modulatory role for the vmPFC in counterfactual processing. These findings not only deepen mechanistic understanding of anxiety-related decision-making but also provide empirical support for computational model-guided neuromodulation, thus marking a crucial step from basic research to clinical translation. Furthermore, our results provide insight into the development of multimodal clinical interventions that have the potential to enhance therapeutic efficacy in trait anxiety cases by integrating cognitive training and neuromodulation therapy.

## Supporting information

10.1017/S0033291726103882.sm001 Ai et al. supplementary material Ai et al. supplementary material

## Supplementary Material

The supplementary material for this article can be found at http://doi.org/10.1017/S0033291726103882.

## Table 1. Long description

The table has eight columns and seven rows. The first column lists variants: Sex (M forward slash F), Age, BD I, SA I, TA I, and BIS dash 11. For Experiment 1, columns are H A, L A, t or chi-squared, and p. For Experiment 2, columns are HA C, HA S, t or chi-squared, and *p*. For Sex, Experiment 1: HA is 20 forward slash 30, LA is 24 forward slash 26, t or chi-squared is 0.65, *p* is 0.42. Experiment 2: HAC is 13 forward slash 17, HAS is 16 forward slash 15, t or chi-squared is 0.56, *p* is 0.52. For Age, Experiment 1: HA is 20.14 plus or minus 1.59, LA is 19.82 plus or minus 1.37, t or chi-squared is minus 1.08, *p* is 0.28. Experiment 2: HAC is 19.90 plus or minus 1.58, HAS is 20.61 plus or minus 2.16, t or chi-squared is minus 1.47, *p* is 0.15. For BD I, Experiment 1: HA is 11.42 plus or minus 7.41, LA is 3.16 plus or minus 2.96, t or chi-squared is minus 7.32, *p* is less than 0.001. Experiment 2: HAC is 14.53 plus or minus 7.95, HAS is 15.19 plus or minus 6.65, t or chi-squared is minus 0.35, *p* is 0.73. For SA I, Experiment 1: HA is 43.90 plus or minus 9.52, LA is 30.58 plus or minus 5.74, t or chi-squared is minus 8.48, *p* is less than 0.001. Experiment 2: HAC is 44.70 plus or minus 5.82, HAS is 45.19 plus or minus 9.56, t or chi-squared is minus 0.24, *p* is 0.81. For TA I, Experiment 1: HA is 48.80 plus or minus 6.70, LA is 33.34 plus or minus 4.43, t or chi-squared is minus 13.61, *p* is less than 0.001. Experiment 2: HAC is 53.80 plus or minus 4.65, HAS is 54.94 plus or minus 5.05, t or chi-squared is minus 0.91, *p* is 0.37. For BIS dash 11, Experiment 1: HA is 58.18 plus or minus 8.39, LA is 56.08 plus or minus 7.31, t or chi-squared is minus 1.34, *p* is 0.19. Experiment 2: HAC is 60.53 plus or minus 8.18, HAS is 62.61 plus or minus 9.84, t or chi-squared is minus 0.9, *p* is 0.37. Notes: HAC is high anxiety cathodal stimulus group, HAS is high anxiety sham stimulus group, BDI is Beck Depression Inventory score, SAI is State Anxiety Inventory score, TAI is Trait Anxiety Inventory score, BIS dash 11 is Impulsivity Inventory score.

Navigate back to Table 1..

## Figure 1. Long description

Top row, left to right: Panel A shows RatingBefore versus Obtained outcome for HA (red) and LA (blue) groups, both increasing linearly from negative to positive outcomes, with HA slightly lower; triple asterisk marks significance. Panel B plots RatingBefore against chance counterfactual, both groups increasing linearly, triple asterisk for significance. Panel C shows RatingAfter versus obtained outcome, both groups increase linearly, double asterisk for significance. Panel D plots RatingAfter against agent counterfactual, both groups increase linearly, triple asterisk for significance. Bottom row, left to right: Panel E shows probability of choosing wheel 1 (P(wheel1)) versus expected value (e), both groups show a sigmoidal increase, triple asterisk for significance. Panel F plots P(wheel1) against risk variance (v), both groups decrease linearly, triple asterisk for significance. Panel G shows P(wheel1) versus anticipated regret (r), both groups increase linearly, triple asterisk for significance. All panels display HA and LA group lines with shaded confidence intervals, and axis ranges are labeled numerically.

Navigate back to Figure 1..

## Figure 2. Long description

Panel A, at the top left, is a schematic top-down view of a head with labeled electrode sites. Red circles mark F3, F4, and Fz as return electrode positions, and Fpz as the target electrode, following the 10-20 EEG system. Panel B, to the right, contains eight 3D brain renderings in two rows. The top row shows dorsal and lateral views, while the bottom row shows sagittal and coronal slices. Color gradients from blue to red represent electric field magnitude (magnE), with red indicating higher intensity, as per the scale bar ranging from 0 to 0.263. Panel C, at the bottom, displays three brain surface renderings. The left and center images show the distribution of E_normal, with blue for negative (cathodal) and red for positive (anodal) values, according to the scale from -0.161 to 0.161. The rightmost image shows a top-down view emphasizing the spatial distribution of polarity across the cortical surface.

Navigate back to Figure 2..

## Figure 3. Long description

The layout consists of four panels on affective ratings (A to D) in the top two rows and three panels on decision making (E to G) in the bottom row. Panel A shows Rating Before versus Obtained outcome for H A and H A C groups under Partial feedback, with both groups showing a strong positive linear trend, significance marked by three asterisks. Panel B plots Rating Before against chance counterfactual, again with a strong positive linear trend and three asterisks for significance. Panel C shows Rating After versus Obtained outcome for Complete feedback, with a positive trend but labeled N S for nonsignificant. Panel D plots Rating After against agent counterfactual, also positive and labeled NS. In the Decision Making section, Panel E shows P open parenthesis wheel 1 close parenthesis versus e (expected value), with a sigmoidal curve and NS. Panel F plots P open parenthesis wheel 1 close parenthesis against v (variance), showing a negative slope with three asterisks for significance and group differences in shading. Panel G shows P open parenthesis wheel 1 close parenthesis versus r (regret), with a positive trend and one asterisk for significance. All panels use color coding for HA and HAC groups, and shaded regions indicate confidence intervals.

Navigate back to Figure 3..
