# Supplementary material for: A causal role of the ventromedial prefrontal cortex in regulating anticipated regret and risk behaviors in trait anxiety
Source: Psychol Med. 2026 Jun 1;56:e173. doi: 10.1017/S0033291726103882 (PMC13234527; doi:10.1017/S0033291726103882)
Supplement: Ai et al. supplementary material [file S0033291726103882sup001.docx]

**Sensitivity analyses**

We performed sensitivity analyses using a stricter criterion (top/bottom 27% of STAI-T scores; n = 29 per group) and a continuous predictor approach to test the robustness of our results. Affective results remained consistent with the primary analysis: High-anxiety (HA) participants exhibited significantly amplified emotional responses to outcomes, chance counterfactuals, and agent counterfactuals compared to low-anxiety (LA) participants (all interaction *ps* < 0.001; see Table S8). Regarding decision-making, the HA group showed stronger modulation by expected value (EV) and risk (V) (interaction: EV, *p* = 0.006; V, *p* < 0.001). Although the interaction with regret was not significant in the categorical split (*p* = 0.16), continuous analysis revealed a trend-level association (*p* = 0.08) that was directionally consistent with the main results of Experiment 1 (Table S9-S10).

[**Supplementary**](link:supplementary) [**materials**](link:materials)


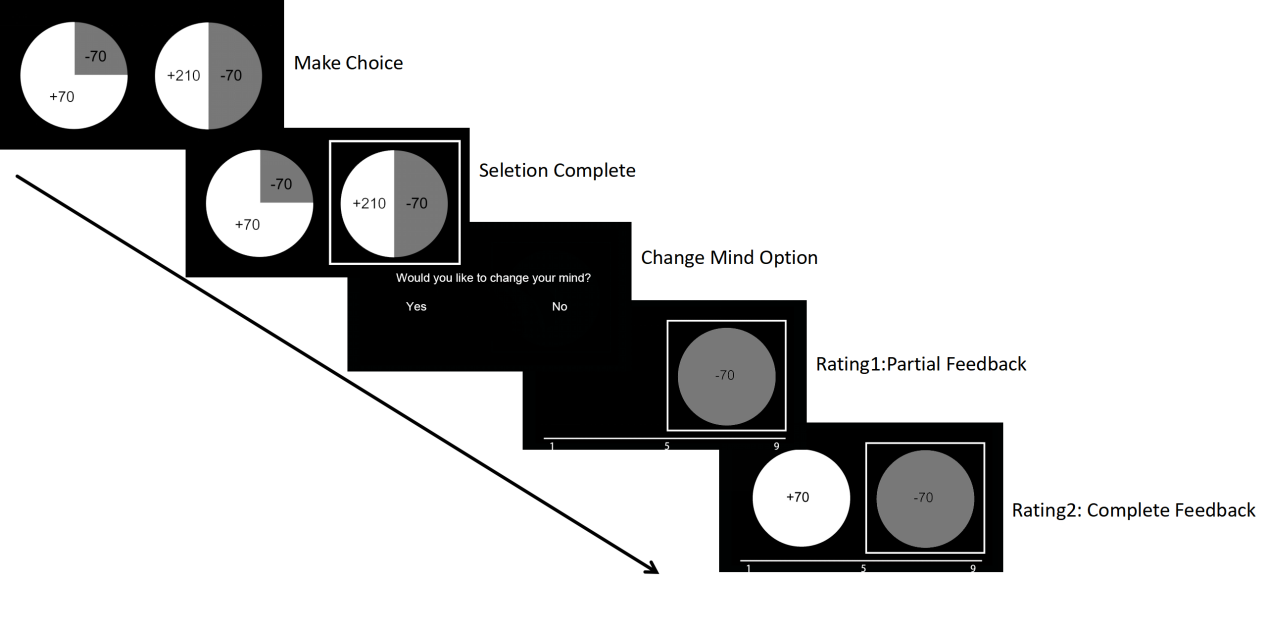


Fig.S1. Schematic of experimental task


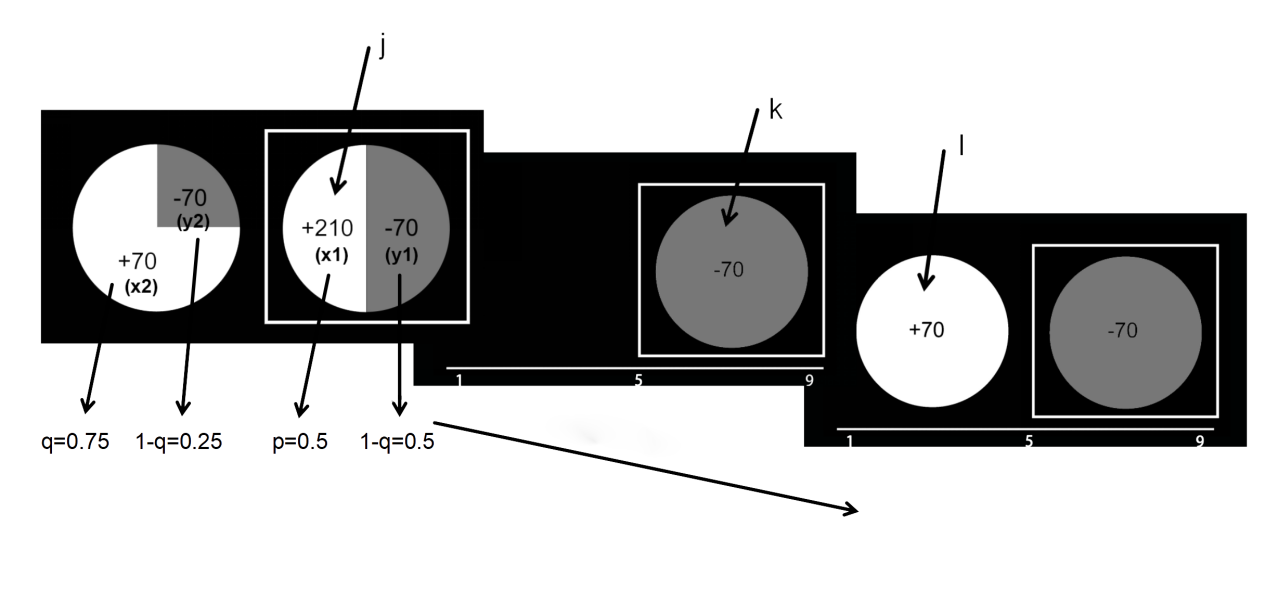


Fig.S2. Rating regression parameters: obtained outcome = k; chance counterfactual = k - j; agent counterfactual = k -l;and decision making model notation: outcome x is always the larger (x1 > y1 and x2 > y2)


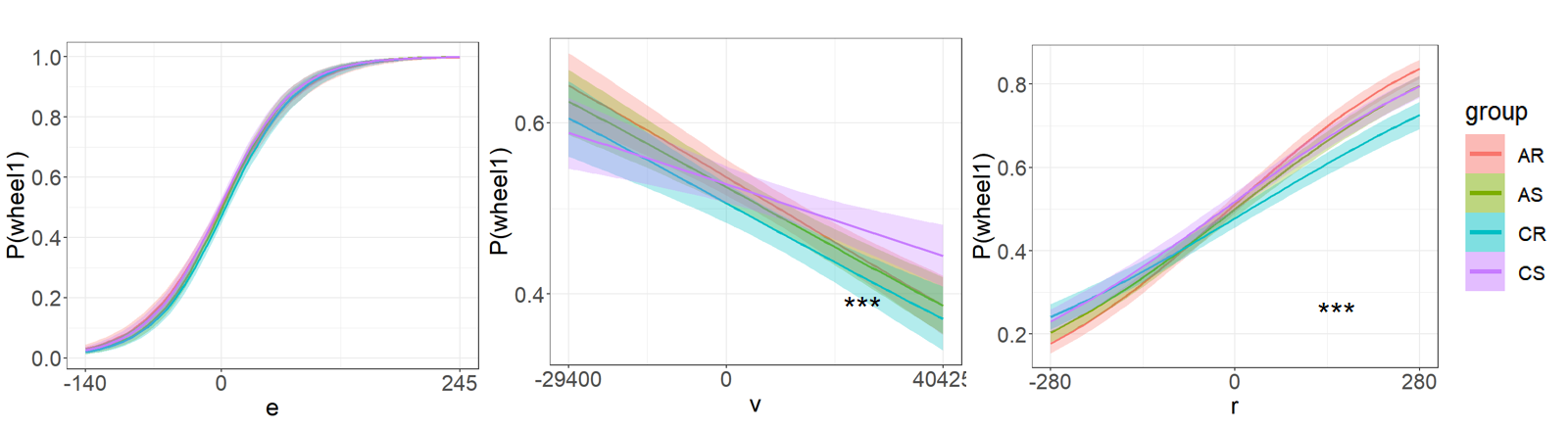


Fig.S3. The logit model-predicted probability of choosing wheel 1 at varying levels of expected value (e), risk variance (v) and anticipated regret (r)..

AR = Anodal group; AS = Anodal Sham group; CR = Cathodal group; CS = Cathodal Sham group. **p* < 0.05, ***p* < 0.01, ****p* < 0.001, NS = nonsignificant.

| Table S1 Decision-making behavioral model in study 1 | | | | | | |
| --- | --- | --- | --- | --- | --- | --- |
| Predictors | Estimate | SE | 95% CI | | z | p |
| (A) Choice model for all subjects (choice ~ e + v + r + group: e + group: v + group: r + (1 \| Subject)) | | | | | | |
| (Intercept) | 0.32 | 0.04 | 0.05 | 0.21 | 7.46 | < 0.001*** |
| e | 0.03 | 0.00 | 1.36 | 1.67 | 19.10 | < 0.001*** |
| v | 0.00 | 0.00 | -0.70 | -0.52 | -15.99 | < 0.001*** |
| r | 0.00 | 0.00 | 0.66 | 0.82 | 17.84 | < 0.001*** |
| e:group | -0.01 | 0.00 | -0.57 | -0.16 | -3.41 | < 0.001*** |
| v:group | 0.00 | 0.00 | 0.18 | 0.42 | 5.33 | < 0.001*** |
| r:group | 0.00 | 0.00 | -0.32 | -0.09 | -3.51 | < 0.001*** |
| 100 subjects, 7996 trials. Log likelihood: -4264.9 | | | | | | |
|  | | | | | | |
| (A) Choice model for anxiety groups (Choice ~ e + v + r + (1\|Subject)) in study 1 | | | | | | |
| (Intercept) | 0.40 | 0.06 | 0.03 | 0.27 | 6.54 | < 0.001*** |
| e | 0.03 | 0.00 | 1.36 | 1.68 | 19.05 | < 0.001*** |
| v | 0.00 | 0.00 | -0.70 | -0.52 | -13.54 | < 0.001*** |
| r | 0.00 | 0.00 | 0.66 | 0.83 | 17.87 | < 0.001*** |
| 50 subjects, 3996 trials. Log likelihood: -2015.5 | | | | | | |
|  | | | | | | |
| (B) Choice model for the control group (Choice ~ e + v + r + (1\|Subject)) in study 1 | | | | | | |
| (Intercept) | 0.24 | 0.06 | 0.01 | 0.23 | 4.17 | < 0.001*** |
| e | 0.02 | 0.00 | 1.02 | 1.28 | 17.54 | < 0.001*** |
| v | 0.00 | 0.00 | -0.39 | -0.23 | -7.59 | < 0.001*** |
| r | 0.00 | 0.00 | 0.46 | 0.61 | 13.89 | < 0.001*** |
| 50 subjects, 4000 trials. Log likelihood: -2249.3 | | | | | | |
| Note: e = expected value, v = risk variance, r = expected regret; 95 per cent CI calculated by converting e, v and r to standardized scores due to proportionality, below. | | | | | | |

| Table S2 Model of decision-making behaviour after controlling for depression levels in experiment 1 | | | | | | |
| --- | --- | --- | --- | --- | --- | --- |
| Predictors | Estimate | SE | 95% CI | | z | p |
| (A) Choice model for all subjects (choice ~ e + v + r + group: e + group: v + group: r + (1 \| Subject)) | | | | | | |
| (Intercept) | 0.33 | 0.06 | 0.05 | 0.27 | 5.78 | 0.003** |
| e | 0.03 | 0.00 | 1.28 | 1.68 | 14.65 | < 0.001*** |
| v | 0.00 | 0.00 | -0.61 | -0.39 | -10.54 | < 0.001*** |
| r | 0.00 | 0.00 | 0.62 | 0.83 | 13.69 | < 0.001*** |
| e:group | 0.00 | 0.00 | -0.35 | 0.20 | -0.49 | 0.63 |
| v:group | 0.00 | 0.00 | -0.03 | 0.28 | 2.02 | 0.04* |
| r:group | 0.00 | 0.00 | -0.41 | -0.12 | -3.61 | < 0.001*** |
| 60 subjects, 4796 trials. Log likelihood: -2541.9 | | | | | | |
|  | | | | | | |
| (A) Choice model for anxiety groups (Choice ~ e + v + r + (1\|Subject)) in study 1 | | | | | | |
| (Intercept) | 0.31 | 0.09 | -0.06 | 0.28 | 3.58 | < 0.001*** |
| e | 0.03 | 0.00 | 1.28 | 1.68 | 14.55 | < 0.001*** |
| v | 0.00 | 0.00 | -0.61 | -0.39 | -8.71 | < 0.001*** |
| r | 0.00 | 0.00 | 0.62 | 0.84 | 13.66 | < 0.001*** |
| 30 subjects, 2396 trials. Log likelihood: -1223.1 | | | | | | |
|  | | | | | | |
| (B)Choice model for the control group (Choice ~ e + v + r + (1\|Subject)) in study 1 | | | | | | |
| (Intercept) | 0.34 | 0.07 | 0.06 | 0.34 | 4.71 | < 0.001*** |
| e | 0.03 | 0.00 | 1.22 | 1.59 | 14.76 | < 0.001*** |
| v | 0.00 | 0.00 | -0.48 | -0.27 | -6.92 | < 0.001*** |
| r | 0.00 | 0.00 | 0.37 | 0.56 | 9.28 | < 0.001*** |
| 30 subjects, 2400 trials. Log likelihood: -1317.9 | | | | | | |

| Table S3 Decision Making Behavioral Models in experiment 2 | | | | | | |
| --- | --- | --- | --- | --- | --- | --- |
| Predictors | Estimate | SE | 95% CI | | z | p |
| (A) Choice model for all subjects (choice ~ e + v + r + group: e + group: v + group: r + (1 \| Subject)) | | | | | | |
| (Intercept) | 0.24 | 0.06 | 0.13 | 0.35 | 4.19 | < 0.001*** |
| e | 0.03 | 0.00 | 0.02 | 0.03 | 14.54 | < 0.001*** |
| v | 0.00 | 0.00 | 0.00 | 0.00 | -4.63 | < 0.001*** |
| r | 0.00 | 0.00 | 0.00 | 0.00 | 10.88 | < 0.001*** |
| e:group | 0.00 | 0.00 | -0.01 | 0.00 | -0.41 | 0.69 |
| v:group | 0.00 | 0.00 | 0.00 | 0.00 | -3.50 | < 0.001*** |
| r:group | 0.00 | 0.00 | 0.00 | 0.00 | 2.36 | < 0.05* |
| 61 subjects, 4845 trials. Log likelihood: -1287.3 | | | | | | |
|  | | | | | | |
| (A) Choice model for the HA group (Choice ~ e + v + r + (1\|Subject)) in study 2 | | | | | | |
| (Intercept) | 0.29 | 0.07 | 0.16 | 0.43 | 4.21 | < 0.001*** |
| e | 0.03 | 0.00 | 0.02 | 0.03 | 14.28 | < 0.001*** |
| v | 0.00 | 0.00 | 0.00 | 0.00 | -8.24 | < 0.001*** |
| r | 0.00 | 0.00 | 0.00 | 0.00 | 13.95 | < 0.001*** |
| 31 subjects, 2460 trials. Log likelihood: -1285.7 | | | | | | |
|  | | | | | | |
| (B) Choice model for the HAC group (Choice ~ e + v + r + (1\|Subject)) in study 2 | | | | | | |
| (Intercept) | 0.12 | 0.09 | -0.05 | 0.28 | 1.36 | < 0.001*** |
| e | 1.40 | 0.10 | 1.21 | 1.59 | 14.45 | < 0.001*** |
| v | -0.21 | 0.05 | -0.31 | -0.10 | -3.92 | < 0.001*** |
| r | 0.55 | 0.05 | 0.45 | 0.65 | 10.82 | < 0.001*** |
| 30 subjects, 2385 trials. Log likelihood: -1287.3 | | | | | | |
| Note: e = expected value, v = risk variance, r = expected regret; 95 per cent CI calculated by converting e, v and r to standardized scores due to proportionality, below. | | | | | | |

| Table S4 Correlation analysis of key variables (*n* = 50) in experiment 1 | | | | | | | | | | | | | | |
| --- | --- | --- | --- | --- | --- | --- | --- | --- | --- | --- | --- | --- | --- | --- |
| Key Variables | age | BDI | SAI | TAI | BIS | Numbers of change | Total task scores | chance_CT | K1 | agent_CT | K2 | e | v | r |
| age | 1.00 | 0.04 | 0.07 | 0.05 | -0.26 | -0.24 | 0.09 | 0.15 | 0.11 | -0.03 | -0.06 | 0.16 | -0.04 | 0.06 |
| BDI |  | 1.00 | 0.61** | 0.69** | 0.17 | -0.06 | 0.21 | 0.08 | 0.07 | 0.14 | 0.16 | -0.11 | -0.15 | -0.08 |
| SAI |  |  | 1.00 | 0.76** | 0.15 | 0.08 | 0.10 | 0.01 | 0.03 | 0.13 | 0.24 | -0.14 | -0.21 | -0.04 |
| TAI |  |  |  | 1.00 | 0.24 | 0.09 | 0.07 | 0.06 | 0.08 | 0.17 | 0.19 | -0.04 | -0.20 | -0.18 |
| BIS |  |  |  |  | 1.00 | 0.26 | -0.20 | 0.08 | 0.05 | 0.10 | 0.02 | -0.19 | -0.13 | -0.13 |
| Numbers of changes |  |  |  |  |  | 1.00 | 0.06 | 0.00 | 0.04 | 0.22 | 0.20 | -0.46** | -0.42** | -0.30* |
| Total task scores |  |  |  |  |  |  | 1.00 | 0.00 | 0.00 | 0.13 | 0.15 | 0.07 | -0.18 | 0.37** |
| chance_CT |  |  |  |  |  |  |  | 1.00 | 0.98** | 0.71** | 0.62** | 0.09 | -0.03 | -0.11 |
| K1 |  |  |  |  |  |  |  |  | 1.00 | 0.69** | 0.66** | 0.02 | -0.07 | -0.15 |
| agent_CT |  |  |  |  |  |  |  |  |  | 1.00 | 0.73** | -0.10 | -0.28* | -0.24 |
| K2 |  |  |  |  |  |  |  |  |  |  | 1.00 | -0.05 | -0.12 | -0.31* |
| e |  |  |  |  |  |  |  |  |  |  |  | 1.00 | 0.55** | 0.21 |
| v |  |  |  |  |  |  |  |  |  |  |  |  | 1.00 | 0.10 |
| r |  |  |  |  |  |  |  |  |  |  |  |  |  | 1.00 |
| Note: chance_CF = chance counterfactual, agent_CF = agent counterfactual, K1 = results obtained (partial feedback), K2 = results obtained (full feedback); ***p < 0.001, **p < 0.01, *p < 0.05, below. | | | | | | | | | | | | | | |

| Table S5 Correlation analysis of key variables (*n* = 31) in experiment 2 | | | | | | | | | | | | | | |
| --- | --- | --- | --- | --- | --- | --- | --- | --- | --- | --- | --- | --- | --- | --- |
| Key Variables | age | BDI | SAI | TAI | BIS | Numbers of change | Total task scores | chance_CT | K1 | agent_CT | K2 | e | v | r |
| age | 1.00 | 0.50 | 0.71 | 0.59 | 0.52 | 0.40 | 0.24 | 0.88 | 0.80 | 0.36 | 0.25 | 0.09 | 0.76 | 0.75 |
| BDI |  | 1.00 | 0.00*** | 0.85 | 0.15 | 0.92 | 0.61 | 0.39 | 0.31 | 0.46 | 0.34 | 0.46 | 0.14 | 0.34 |
| SAI |  |  | 1.00 | 0.58 | 0.55 | 0.92 | 0.73 | 0.01** | 0.01** | 0.02* | 0.01* | 0.17 | 0.59 | 0.65 |
| TAI |  |  |  | 1.00 | 0.45 | 0.07 | 0.57 | 0.76 | 0.87 | 0.60 | 0.73 | 0.61 | 0.96 | 0.92 |
| BIS |  |  |  |  | 1.00 | 0.63 | 0.75 | 0.25 | 0.26 | 0.06 | 0.07 | 0.76 | 0.80 | 0.19 |
| Numbers of changes |  |  |  |  |  | 1.00 | 0.07 | 0.84 | 0.82 | 0.73 | 0.71 | 0.62 | 0.07 | 0.63 |
| Total task scores |  |  |  |  |  |  | 1.00 | 0.67 | 0.71 | 0.48 | 0.94 | 0.32 | 0.39 | 0.00*** |
| chance_CT |  |  |  |  |  |  |  | 1.00 | 0.00*** | 0.00*** | 0.00*** | 0.85 | 0.41 | 0.57 |
| K1 |  |  |  |  |  |  |  |  | 1.00 | 0.00*** | 0.00*** | 0.96 | 0.33 | 0.60 |
| agent_CT |  |  |  |  |  |  |  |  |  | 1.00 | 0.00*** | 0.93 | 0.40 | 0.70 |
| K2 |  |  |  |  |  |  |  |  |  |  | 1.00 | 0.82 | 0.49 | 0.51 |
| e |  |  |  |  |  |  |  |  |  |  |  | 1.00 | 0.11 | 0.31 |
| v |  |  |  |  |  |  |  |  |  |  |  |  | 1.00 | 0.07 |
| r |  |  |  |  |  |  |  |  |  |  |  |  |  | 1.00 |
| Note: chance_CF = chance counterfactual, agent_CF = agent counterfactual, K1 = results obtained (partial feedback), K2 = results obtained (full feedback); ***p < 0.001, **p < 0.01, *p < 0.05, below. | | | | | | | | | | | | | | |

| Table S6 Decision Response Time Model in experiment 1 | | | | | | |
| --- | --- | --- | --- | --- | --- | --- |
| Predictors | Estimate | SE | 95% CI | | z | p |
| intercept | 3.55 | 0.12 | 3.31 | 3.79 | 29.11 | <0.001*** |
| e_abs | -0.01 | 0.00 | -0.01 | 0.00 | -7.02 | <0.001*** |
| v_abs | 0.00 | 0.00 | 0.00 | 0.00 | -6.75 | <0.001*** |
| r_abs | 0.00 | 0.00 | 0.00 | 0.00 | -4.30 | <0.001*** |
| e_abs*group | 0.00 | 0.00 | 0.00 | 0.00 | 0.40 | 0.69 |
| v_abs*group | 0.00 | 0.00 | 0.00 | 0.00 | 1.32 | 0.19 |
| r_abs*group | 0.00 | 0.00 | 0.00 | 0.00 | 0.10 | 0.92 |
| 100 subjects, 7996 observations. Log likelihood: -16685.39 | | | | | | |
| Note: abs are the corresponding absolute values. | | | | | | |

| Table S7 Decision Response Time Model in experiment 2 | | | | | | |
| --- | --- | --- | --- | --- | --- | --- |
| Predictors | Estimate | SE | 95% CI | | z | p |
| intercept | 0.24 | 0.06 | 0.13 | 0.35 | 4.19 | <0.001*** |
| e_abs | 0.03 | 0.00 | 0.02 | 0.03 | 14.33 | <0.001*** |
| v_abs | 0.00 | 0.00 | 0.00 | 0.00 | -9.48 | <0.001*** |
| r_abs | 0.00 | 0.00 | 0.00 | 0.00 | 13.98 | <0.001*** |
| e_abs*group | 0.00 | 0.00 | 0.00 | 0.01 | 0.41 | 0.68 |
| v_abs*group | 0.00 | 0.00 | 0.00 | 0.00 | 3.55 | <0.001*** |
| r_abs*group | 0.00 | 0.00 | 0.00 | 0.00 | -2.36 | 0.0184* |
| 61 subjects, 4845 observations. Log likelihood: -2574.4 | | | | | | |
| Note: abs are the corresponding absolute values. | | | | | | |

Table S8 Descriptive statistics and scores on each scale (M ± SD) for groups based on 27% cutoff

| Variants | 27% cut off (HA = 29, LA = 29) | | | |
| --- | --- | --- | --- | --- |
|  | HA | LA | t / χ2 | p |
| Sex (M / F) | 11 / 18 | 14 / 15 | 0.63 | 0.43 |
| Age | 20.24 ± 1.81 | 19.90 ± 1.57 | 0.78 | 0.44 |
| BDI | 14.28 ± 7.31 | 2.21 ± 1.88 | 8.61 | < 0.001 |
| SAI | 48.97 ± 9.15 | 27.79 ± 4.12 | 11.37 | < 0.001 |
| TAI | 53.59 ± 4.88 | 29.97 ± 3.20 | 21.79 | < 0.001 |
| BIS-11 | 60.41 ± 9.73 | 54.62 ± 8.17 | 2.46 | 0.02 |

| Table S9 Decision-making behavioral model results using the 27% cutoff | | | | | | |
| --- | --- | --- | --- | --- | --- | --- |
| Predictors | Estimate | SE | 95% CI | | z | p |
| (A) Choice model for all subjects (choice ~ e + v + r + group: e + group: v + group: r + (1 \| Subject)) | | | | | | |
| (Intercept) | 0.20 | 0.05 | 0.10 | 0.29 | 4.21 | < 0.001*** |
| e | 1.59 | 0.11 | 1.39 | 1.80 | 14.94 | < 0.001*** |
| v | -0.66 | 0.06 | -0.77 | -0.54 | -11.24 | < 0.001*** |
| r | 0.63 | 0.05 | 0.53 | 0.74 | 11.70 | < 0.001*** |
| e:group | -0.38 | 0.14 | -0.65 | -0.11 | -2.74 | 0.006** |
| v:group | 0.35 | 0.08 | 0.20 | 0.51 | 4.50 | < 0.001*** |
| r:group | -0.10 | 0.07 | -0.25 | 0.04 | -1.41 | 0.16 |
| 58 subjects, 4639 trials. Log likelihood: -2478.9 | | | | | | |
|  | | | | | | |
| (A) Choice model for HA group (choice ~ e + v + r + (1\|Subject)) | | | | | | |
| (Intercept) | 0.21 | 0.07 | 0.07 | 0.35 | 3.02 | 0.003** |
| e | 1.59 | 0.11 | 1.39 | 1.81 | 14.85 | < 0.001*** |
| v | -0.66 | 0.06 | -0.78 | -0.55 | -11.22 | < 0.001*** |
| r | 0.63 | 0.05 | 0.53 | 0.74 | 11.69 | < 0.001*** |
| 29 subjects, 2319 trials. Log likelihood: -1182.4 | | | | | | |
|  | | | | | | |
| (B) Choice model for LA group (choice ~ e + v + r + (1\|Subject)) | | | | | | |
| (Intercept) | 0.19 | 0.06 | 0.06 | 0.31 | 2.94 | 0.003** |
| e | 1.21 | 0.09 | 1.04 | 1.39 | 13.60 | < 0.001*** |
| v | -0.31 | 0.05 | -0.41 | -0.20 | -5.86 | < 0.001*** |
| r | 0.53 | 0.05 | 0.43 | 0.63 | 10.41 | < 0.001*** |
| 29 subjects, 2320 trials. Log likelihood: -1295.1 | | | | | | |
| Note: e = expected value, v = risk variance, r = expected regret; 95 per cent CI calculated by converting e, v and r to standardized scores due to proportionality, below. | | | | | | |

| Table S10 Decision-making behavioral model results with STAI-T scores as a continuous predictor | | | | | | |
| --- | --- | --- | --- | --- | --- | --- |
| Predictors | Estimate | SE | 95% CI | | z | p |
| (A) Choice model for all subjects (choice ~ e + v + r + TAI + e: TAI + v: TAI + r: TAI + (1 \| Subject)) | | | | | | |
| (Intercept) | 0.13 | 0.04 | 0.05 | 0.20 | 3.38 | < 0.001*** |
| e | 1.30 | 0.05 | 1.20 | 1.40 | 26.31 | < 0.001*** |
| v | -0.43 | 0.03 | -0.49 | -0.38 | -15.04 | < 0.001*** |
| r | 0.64 | 0.03 | 0.59 | 0.70 | 23.27 | < 0.001*** |
| TAI | 0.00 | 0.04 | -0.07 | 0.08 | 0.01 | 0.99 |
| e:TAI | 0.13 | 0.05 | 0.03 | 0.22 | 2.52 | 0.01* |
| v:TAI | -0.15 | 0.03 | -0.21 | -0.09 | -5.19 | < 0.001*** |
| r:TAI | 0.05 | 0.03 | -0.01 | 0.10 | 1.75 | 0.08 |
| 106 subjects, 8475 trials. Log likelihood: -4533.0 | | | | | | |
| Note: e = expected value, v = risk variance, r = expected regret; 95 per cent CI calculated by converting e, v and r to standardized scores due to proportionality, below. | | | | | | |

Table S11 Descriptive statistics and scores on each scale (M ± SD) for the healthy individuals

|  | Anodal | Anodal Sham | Cathodal | Cathodal Sham | F/χ^2^ | *p* |
| --- | --- | --- | --- | --- | --- | --- |
| N | 33 | 36 | 28 | 32 |  |  |
| Sex(male/female) | 18/15 | 18/18 | 17/11 | 16/16 | 0.94 | 0.82 |
| Age | 21.06±1.94 | 21.58±2.27 | 21.44±2.40 | 21.46±2.83 | 0.38 | 0.77 |
| BDI | 3.36±3.72 | 2.83±2.99 | 2.59±2.65 | 3.29±3.17 | 0.34 | 0.79 |
| RRS | 36.70±5.53 | 37.92±4.99 | 37.47±5.38 | 37.49±4.98 | 0.32 | 0.81 |
| SAI | 31.19±5.42 | 33.08±6.16 | 32.25±6.58 | 31.34±5.44 | 1.16 | 0.33 |
| TAI | 38.55±7.50 | 38.56±7.32 | 34.94±6.72 | 34.11±6.41 | 2.38 | 0.07* |
| BIS | 53.33±7.27 | 56.92±9.45 | 55.44±6.24 | 53.20±10.45 | 1.69 | 0.17 |
